# The NIEHS Environmental Health Sciences Data Resource Portal: Placing Advanced Technologies in Service to Vulnerable Communities

**DOI:** 10.1289/ehp.9817

**Published:** 2007-01-22

**Authors:** Keith Pezzoli, Robert Tukey, Hiram Sarabia, Ilya Zaslavsky, Marie Lynn Miranda, William A. Suk, Abel Lin, Mark Ellisman

**Affiliations:** 1 Urban Studies and Planning Program; 2 Department of Pharmacology; 3 Department of Chemistry and Biochemistry and; 4 San Diego Supercomputer Center, University of California San Diego, La Jolla, California, USA; 5 Nicholas School of the Environment and Earth Sciences, Duke University, Durham, North Carolina, USA; 6 National Institute of Environmental Health Sciences, National Institutes of Health, Department of Health and Human Services, Research Triangle Park, North Carolina, USA; 7 National Center for Microscopy and Imaging Research, Center for Research in Biological Systems and the Department of Neurosciences, University of California San Diego, La Jolla, California, USA

**Keywords:** community-linked research, cyberinfrastructure, disaster, environmental justice, GIS, grid, health disparities, integrative research, Katrina, telescience

## Abstract

**Background:**

Two devastating hurricanes ripped across the Gulf Coast of the United States during 2005. The effects of Hurricane Katrina were especially severe: The human and environmental health impacts on New Orleans, Louisiana, and other Gulf Coast communities will be felt for decades to come. The Federal Emergency Management Agency (FEMA) estimates that Katrina’s destruction disrupted the lives of roughly 650,000 Americans. Over 1,300 people died. The projected economic costs for recovery and reconstruction are likely to exceed $125 billion.

**Objectives:**

The NIEHS (National Institute of Environmental Health Sciences) Portal aims to provide decision makers with the data, information, and the tools they need to *a*) monitor human and environmental health impacts of disasters; *b*) assess and reduce human exposures to contaminants; and *c*) develop science-based remediation, rebuilding, and repopulation strategies.

**Methods:**

The NIEHS Portal combines advances in geographic information systems (GIS), data mining/integration, and visualization technologies through new forms of grid-based (distributed, web-accessible) cyberinfrastructure.

**Results:**

The scale and complexity of the problems presented by Hurricane Katrina made it evident that no stakeholder alone could tackle them and that there is a need for greater collaboration. The NIEHS Portal provides a collaboration-enabling, information-laden base necessary to respond to environmental health concerns in the Gulf Coast region while advancing integrative multidisciplinary research.

**Conclusions:**

The NIEHS Portal is poised to serve as a national resource to track environmental hazards following natural and man-made disasters, focus medical and environmental response and recovery resources in areas of greatest need, and function as a test bed for technologies that will help advance environmental health sciences research into the modern scientific and computing era.

The 2006–2011 National Institute of Environmental Health Sciences’ (NIEHS) Strategic Plan *New Frontiers in Environmental Sciences and Human Health* (NIEHS 2006c) seeks to “challeng[e] and energiz[e] the scientific community to use environmental health sciences to understand the causes of disease and to improve human health” (National Institutes of Health 2006). The strategic plan emphasizes, among other things, integrative research, community-linked research, and prioritizing environmental factors that are most likely contributing to human disease. Consistent with the goals of the strategic plan, new technologies in information systems, data federation, grid systems, and spatial analytics all hold great promise of enabling integrated science teamwork while helping to disentangle genetic, environmental, and other factors that contribute to the complex etiology of common human diseases. Such systems will provide effective means of knowledge and data sharing among scientists, as well as rapid and efficient approaches for sorting through and analyzing large data sets; thus, these systems will support both the scientific and policy processes while accelerating the rate of knowledge production and discovery.

The need for such systems to address environmental health concerns was made especially apparent when a series of devastating hurricanes ripped across the Gulf Coast of the United States during 2005 (Schwartz 2005). The effects of Hurricane Katrina were especially severe: The human and environmental health impacts on New Orleans, Louisiana, and other Gulf Coast communities will be felt for many decades to come [Daniels et al. 2006; Nates and Moyer 2005; Pastor et al. 2006; Pezzoli et al. 2006; U.S. Environmental Protection Agency (EPA) 2006]. The Federal Emergency Management Agency (FEMA) estimates that Katrina’s destruction (exacerbated by the chronic loss of wetlands, land subsidence, and inadequate infrastructure) disrupted the lives of roughly 650,000 Americans (U.S. Department of Commerce 2006). Over 1,300 people died. The projected economic costs for recovery and reconstruction are unprecedented—likely to exceed $125 billion (Pastor et al. 2006).

The density of industrial operations in New Orleans and the Mississippi Delta before the Katrina disaster combined with the release of contaminants into floodwaters and their transport throughout urbanized areas to give rise to many significant environmental health concerns. At the same time, the flooding of thousands of homes and > 100 schools may have created indoor environments that threaten human respiratory and neurologic health through the growth of mold and other bioaeroallergens. All of these potential exposures took place within the context of high psychosocial stress and, for many, low socioeconomic status—conditions that often exacerbate the effects of adverse environmental exposures (Berube and Katz 2005). Health disparities that result from poverty and inadequate infrastructure raise serious concerns about environmental justice (Pellow and Brulle 2005; Thomson et al. 2006). The scale and complexity of the ensuing environmental health issues made tracking environmental hazards and focusing medical and environmental response and recovery resources in areas of greatest need an obvious priority. A unique opportunity arose to deploy a system to meet these needs, while also creating a test bed for technologies that will help advance environmental health sciences research into the modern scientific and computing era.

## Objectives

As Hurricane Katrina made landfall, teams of NIEHS staff mobilized various relief efforts. Within the Division of Extramural Research and Training, a group of Superfund Basic Research Program (SBRP) grantees pooled intellectual resources to create an internet portal that couples the power of geographic information systems (GIS) and grid technologies. The NIEHS Environmental Health Sciences Data Resource Portal (NIEHS Portal) provides common access and the ability to share information, software, and computer processes across organizational boundaries within a user-friendly, highly customizable environment (NIEHS 2006a).

The NIEHS Portal aims to provide scientists and decision makers with the data, information, and tools they need to *a*) monitor and evaluate human and environmental health impacts in the Gulf Coast Region; *b*) assess and reduce human exposures to contaminants; and *c*) develop science-based remediation, rebuilding, and repopulation strategies. In this article we describe the NIEHS Portal’s aims and prospects for its utilization from analytical and applied perspectives. The NIEHS Portal allows users to access demographic, public health, infrastructure, and environmental data, all of which are geo-referenced. The spatial data sets incorporated into the portal include basic infrastructure data such as roads and electric power plants, potential contaminant-release sources including Superfund and Toxics Release Inventory (TRI) sites, hurricane flooding data, Census data, physiographic data, and remote sensing imagery both pre- and post-Katrina. Creating a unified environmental health GIS, integrating it in a secure web portal, and providing customized research environments for teams of collaborators are the main technical components of the project.

## Context and Methods

The NIEHS Portal represents a proactive response to four trends that, in addition to helping explain the nature of the portal initiative itself, are key to understanding the new direction and priorities spelled out in the NIEHS strategic plan (NIEHS 2006c). The four trends include *a*) the rising magnitude of disasters and vulnerability on a global scale; *b*) the emergence of new modes of knowledge production, integration, and networking; *c*) an increasing emphasis on integrative and community-linked research in the culture and practice of science; and *d*) new investments in exposure biology and gene–environment interaction studies.

### Rising magnitude of disasters and vulnerability on a global scale

The continued function and survival of any human society is dependent, to a significant degree, on its adaptability, resilience, and vulnerability to environmental events (Young et al. 2006). In spite of our increased awareness of environmental hazards and risks, the human, ecologic, and economic costs of disasters continue to rise sharply worldwide. Over the last two decades, disasters including floods, drought, hurricanes, earthquakes, and tsunamis have affected vast regions and claimed nearly a million human lives, indirectly impacted a billion people, and generated damages in the trillions of dollars [Nates and Moyer 2005; U.K. Department for International Development (DFID) 2006]. The effects of these disasters on populations can be explained in part by a growing human footprint in the environment, particularly in vulnerable areas (e.g., low-lying coastal areas, flood plains, steep hillsides), which makes encountering risk and the possibility of exposure to environmental hazards more likely (Nates and Moyer 2005). At the same time, global climate change is expected to increase the frequency and intensity of floods, hurricanes, and wildfires, and lead to sea level change (Barnett et al. 2001; Running 2006; Westerling et al. 2006).

**Figure 1:**
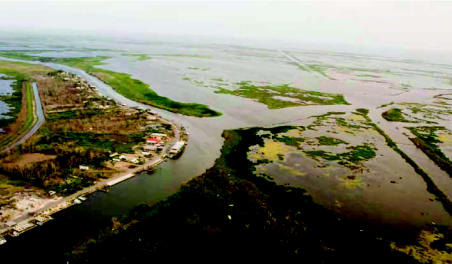
Wetland destruction caused by Hurricane Katrina near New Orleans, April 2006 (photographed by K. Pezzoli).

As globalization accelerates the rate and spatial scale of human–environment interactions, the distinction between natural and man-made disasters becomes blurred. So-called natural disasters are often exacerbated by anthropogenic factors. In the case of Hurricane Katrina, for instance, the development of and loss of low-lying wetlands (Figure 1) greatly diminished a natural ecosystem buffer that would have otherwise provided a greater degree of protection from the storm surge that killed many people and did severe damage to industrial facilities. The later led to a number of chemical spills that exacerbated the damage already caused by the hurricane.

The negative impact of environmental events can be reduced by good planning. For example, this is evident in New Orleans when we compare the impact of flooding in newer neighborhoods built on risk-prone, low-lying wetlands (i.e., New Orleans East) with older sections of the city that the Spanish and French built at safer higher elevations. Similarly, the operation of industrial facilities near densely populated areas can lead to increased hazards. This is exemplified by the Murphy Oil spill in the St. Bernard Parish of New Orleans, where a storage tank at the Meraux Murphy Oil Refinery, adjacent to a heavily populated area, was damaged by flood waters and spilled an estimated 1.05 million gallons of crude oil affecting some 1,800 homes (Pezzoli et al. 2006).

**Figure 2:**
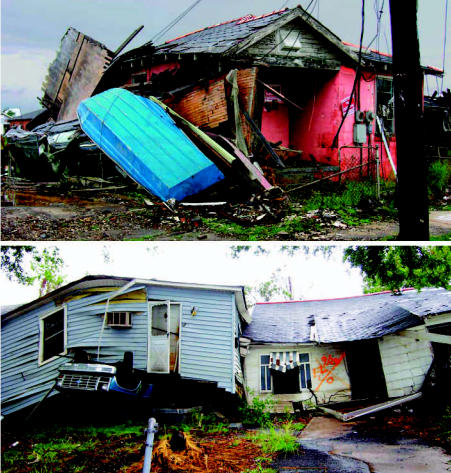
Houses destroyed in Lower Ninth Ward of New Orleans, April 2006 (photographed by K. Pezzoli).

Poverty-stricken communities experience the most severe social and economic impacts of disasters, often with long-lasting and devastating effects (Daniels et al. 2006; Dilley et al. 2005; U.K. DFID 2006). The Lower Ninth Ward, a poverty-stricken part of New Orleans, was utterly devastated (Figure 2). Disasters highlight the interdependence of communities at local, regional, and global scales as well as the importance of building disaster response systems that are flexible, adaptable, and resilient (Daniels et al. 2006).

### New modes of knowledge production and networking

In recent years, information and communications technology experts, in partnership with researchers, have built sophisticated virtual environments that are enabling multidisciplinary and geographically distributed teams to work together more effectively. This includes sharing resources and knowledge and responding to grand challenges, such as those consequent to the hurricanes and floods that devastated the Gulf Coast in 2005. New modes of knowledge production and networking are exemplified by e-science and cyberinfrastructure that involve collaborative use of distributed high-speed networks, high-performance supercomputing, and massive storage to tackle the processing and storage-intensive questions of modern scientific research (Buetow 2005; Hey and Trefethen 2005; Lin et al. 2005a; Mattes et al. 2004). In the same way that early bioinformatics played a crucial role in the completion of the Human Genome Project, the next phase, the Human Proteonome Folding Project, is now using grid-based technologies to elucidate the structure and function of all the proteins encoded by the Human Genome. This grid-based approach promises to provide valuable knowledge for understanding and curing disease. Computing that takes advantage of grid technology can shorten computer time by many orders of magnitude.

The European Council for Nuclear Research (CERN) is one of the world’s leaders in grid development. CERN (2006) defined the grid in these terms:

Whereas the Web is a service for sharing information over the Internet, the Grid is a service for sharing computer power and data storage capacity over the Internet. The Grid goes well beyond simple communication between computers and aims ultimately to turn the global network of computers into one vast computational resource.

One of the best developed examples of grid-based cyberinfrastructure is the Telescience model utilized by the NIEHS Portal. Through collaborative arrangements agreed upon by those developing and using the resource, the Telescience model integrates distributed ultra–high speed networks, supercomputing capacity, and access to massive storage and databases with applications, virtual collaboration spaces, advanced visualization, and remote control of laboratory instruments and field sensors with advanced authentication and authorization features (Lathers et al. 2006; Lin et al. 2005b). This kind of collaborative cyberinfrastructure is essential to build capacity for data mining and data sharing in the environmental health sciences.

Another significant development involves “ontologies”—a kind of knowledge map to facilitate data organization and mining for certain predefined purposes (Noy and Musen 2003; Rubin et al. 2006; Stoeckert et al. 2006). Ontologies or knowledge maps that include formal definitions of terms and their relationship to one another can help to distinguish between irrelevant information and information of interest, while also addressing important issues of communication among members of multidisciplinary science teams (Martone et al. 2004). The NIEHS Portal aims to facilitate, among other things, knowledge networking within communities by creating task- and domain-specific ontologies that can be used for applied purposes. The development of such ontologies must be participatory in so far as it requires a common language that cuts across disciplines and professional boundaries (Spivey 2004).

### Integrative and community-linked research

An important shift in the historical relationship between science and society has taken place over the last few decades, particularly as it pertains to university–community partnerships outside of the traditional university–government–industry triad (Chopyak and Levesque 2002). This has occurred in part because of changes in funding policies of governmental and private agencies that demand greater social accountability from universities and researchers. This is reflected in the community outreach and/or research translation requirements of many long-standing and important sources of funding for university investigators (e.g., National Science Foundation, National Institutes of Health). The relationship between universities and the communities they serve has also continued to evolve. For example, after Hurricane Katrina, Tulane University saw a very significant portion of its research resources refocused to address the challenges facing New Orleans and its communities. The benefits of such community-linked research collaborations can be far reaching for both the researchers and the community because they provide a fertile setting with real-world feedback loops to put ideas and advances in science and technology to the test while addressing community concerns (Anderson et al. 2002; Chopyak and Levesque 2002). In the environmental health sciences, community-based research has an important role to play and is recognized by the NIEHS in its strategic plan (Schwartz et al. 2006).

The NIEHS Portal responds to environmental health issues and concerns facing communities of the Gulf Coast region while providing the cyberinfrastructure necessary to advance integrative research and facilitate community-linked research. In this regard, one important measure of performance for the portal will be the extent to which it contributes toward helping scientists and decision makers address community-based environmental health concerns and issues. Efforts of the NIEHS Portal development team to address community needs have been documented (Pezzoli et al. 2006).

### Exposure biology and gene–environment interaction studies

Progress in the study of exposure biology and gene–environment interactions promises to lead to significant advances in disease prediction, prevention, early detection, and treatment (Acharya et al. 2004; Lee and Chang 2006; Shostak 2005; Weis et al. 2005). In at least two significant ways, the NIEHS Portal provides a platform for developing grid-based technologies that directly address the needs of exposure biology and gene–environment work. First, the NIEHS Portal creates capacity to manage the unprecedented amounts of data anticipated from population-level studies involving genetic analyses, data collection by real-time personal exposure devices, and environmental monitoring. It also enables access to and integration of external, distributed data sources. Second, by integrating distributed high-performance computing and GIS into one virtual online workspace, the NIEHS Portal provides simultaneous capacity to integrate spatio-temporal queries and analyses (of demographic, genetic, and environmental data sets) with modeling and other applications.

### Assembling and implementing the portal technology

**Figure 3:**
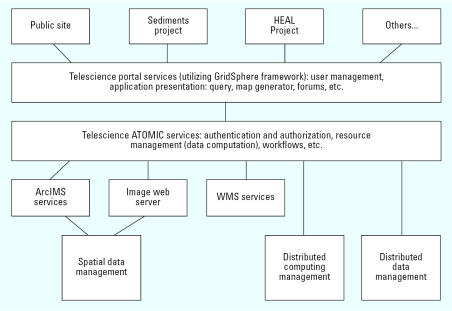
NIEHS Portal system architecture. Abbreviations: ATOMIC, Applications to Middleware Interaction Components; WMS, web mapping service.

The portal’s infrastructure was assembled using components developed within the University of California, San Diego Telescience project (Lin et al. 2005b). The portal-based GIS application is built over a collection of geo-referenced data layers using Microsoft-based ESRI ArcIMS (ESRI, Redlands, CA), ERMapper Image Web Server (ERMapper, San Diego, CA), and open-source solutions for managing spatial databases and large data grid storage space as the core software (Figure 3). In addition, street data are served via a dedicated web mapping service, whereas navigation support is provided via Inxight’s Vizserver (Inxight Software, Inc, Sunnyvale, CA). The user–interface components, including an online map viewer and the StarTree hyperbolic tree visualization application (Inxight Sotware, Inc.), are integrated in a GridSphere-based portal (gridsphere.org), which also supports a variety of browse, query, and collaboration tools (Inxight Software, Inc.). A detailed discussion of the system architecture, specifications, and function is presented by Zaslavsky et al. (2006).

**Figure 4:**
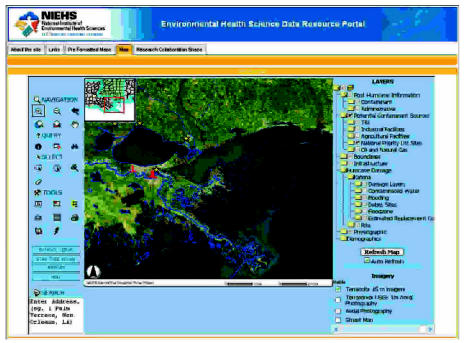
NIEHS Portal GIS.

Within the main portal environment, a GIS application manages the large collection of geo-referenced data. Standard components of the NIEHS Portal also enable local decision makers and communities to contribute data, participate in discussions, and maintain research and collaboration workspaces. The online GIS, a core component of the NIEHS Portal, assembles spatially registered data for Texas, Louisiana, and Mississippi, with higher resolution layers available for areas affected by the two most severe 2005 hurricanes, Katrina and Rita (Figure 4). The information is extracted from publicly available sources and organized in the following data categories:

- Post-hurricane project-related data
- Potential contaminant sources: U.S. EPA TRI facilities (hazardous air pollutants, sources of carcinogens, metals, persistent bioaccumulative toxic substances, and other organic compounds); multiple types of industrial and agricultural facilities, oil and gas facilities (including gas stations, oil and gas wells and platforms, refineries, storage facilities, lube plants, and pipelines) and wastewater treatment plants, among others
- Electric facilities and drinking water intakes
- Hurricane damage: Katrina and Rita damage for all available dates (including impassable bridges and roads, and different degrees of damage), extent of oil spills and water contamination, extent and duration of flooding, debris, and estimated replacement costs
- Demographic data: racial composition and income stratification by census tracts and block groups
- Base map layers: states, counties, and cities; urban areas, streets and landmarks, hydrologic layers, and elevation
- Imagery layers: 1-m aerial photography for New Orleans taken in September 2005, 15-m Terracolor imagery, 1-m Terraserver aerial photographs.

Each of the vector layers—that is, data in the form of points, lines, and polygons—can be downloaded from the portal along with metadata (i.e., details about the data itself, such as who collected it, over what time period, etc.). Although the NIEHS Portal has the potential to bring forward crucial time-sensitive information that might not otherwise be readily available (e.g., types and quantities of hazardous materials generated or stored at an industrial facility), decision makers will still have to rely on trained and experienced professionals to analyze and interpret those data and make intelligent and informed management decisions. There are dangers associated with the misuse of data—a reality facing many information systems. The NIEHS Portal takes this fact into account by including security measures and authentication protocols. Other features of the portal, including clearly documented metadata, are designed to help reduce and manage the risk of data misuse.

**Figure 5:**
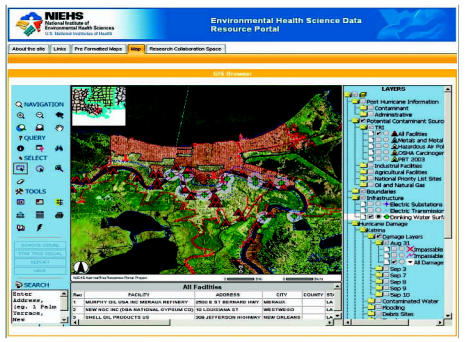
NIEHS Portal GIS showing workspace in which a user selected TRI facilities within 1 mile of drinking water surface intakes.

Users can navigate spatial layers and explore layer metadata using StarTree hyperbolic tree software. In addition to metadata management, the current version of the GridSphere-based online mapping environment provides a range of map navigation, address geocoding, and query and spatial selection tools, including Web Portal, which provides a means for local decision makers and communities to contribute data, participate in discussions, and maintain research and collaboration workspaces. As an example of online-mapping functionality available through the public section of the portal, Figure 5 shows a screen capture of a workspace in which a user selected TRI facilities within 1 mile of drinking water intakes. Password-protected sections of the portal may provide access to additional resources and custom functions. These secure areas of the portal help users collaborate in specific projects.

The NIEHS Portal is a work in progress; it aims to engage users and communities that need to share important information pertinent to an environmental disaster. Customized research environments are identified and developed according to two models: *a*) project-based (the portal team is enlisted by prospective end users to provide information technology support for a particular project); and *b*) field of research [through conversations with multiple teams of investigators, the portal team provides data, knowledge, and tools for a select field of research (e.g., basic and applied studies concerning contaminated sediments)].

In the first model, the portal team is expected to *a*) create a separate space on the portal for the selected project; *b*) sculpt the particular project’s user interface to include only the portal’s most useful existing data layers (i.e., avoid clutter by eliminating layers deemed nonessential); *c*) do some original data preparation/packaging to meet the needs of the particular project; and *d*) provide tools for secure data archiving, sharing, integration, visualization, and analysis. In this model, the portal team becomes (among other things) a service provider helping users meet particular needs (e.g., the need to select a study site).

**Figure 6:**
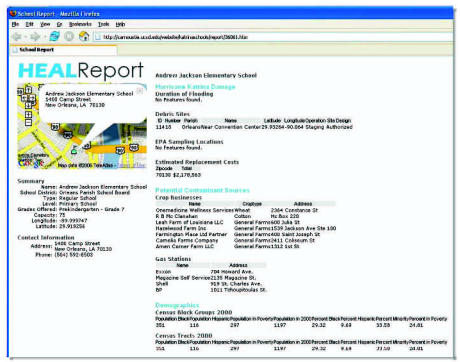
NIEHS Portal HEAL application (NIEHS 2006b).

As an example of the first model of portal applications, the portal team has developed resources to support and is communicating with the Head-Off Environmental Asthma in Louisiana (HEAL) research project. The floods caused by Hurricanes Katrina and Rita left many homes in the Gulf Region inundated, leading to prolific mold growth [according to the Centers for Disease Control and Prevention (CDC), almost 46% of homes inspected had mold growth]. The degradation of indoor/outdoor air quality and disruption of the health care delivery system had significant effects on children living with asthma, which is already the most common chronic disease among children. The goal of the HEAL section of the portal is to support an epidemiologic study and the examination of genetic and environmental factors related to asthma, focusing on the implementation of a case management program for children with asthma in the impacted region. GIS support for the HEAL initiative can help investigators and other stakeholders gauge levels of environmental risk associated with schools that re-opened after the hurricane. GIS helps in this type of field evaluation, given the need to integrate multiple sources of information including data about the populations and households served by those schools, flooding depth and duration, potential environmental exposures, proximity to industrial facilities, proximity to roadways, and sociodemographic data (Figure 6).

Figure 6 illustrates a printable report for a given point location that specifies the location of each school vis-à-vis objects in several other selected layers (e.g., potential contaminant sources, debris sites, duration of flooding). Available metadata at the “feature level” include many more parameters beyond those shown in Figure 6. It is an extensive collection that also includes facility identification information that is available in the original database. Metadata at the “layer level” contains a link to the original data source. In the example shown in Figure 6, there are nine feature-level metadata characteristics for debris sites around a particular school. The debris site shapefile, downloadable from the portal, contains 35 fields, including the purpose and operations at the debris site, its size, land use and ownership, and detailed information on what specific activities or items are permitted on the site. The generation of metadata reports of this sort is not limited to schools. Metadata reports can be created on an as-needed basis by the user for a wide range of particular locations.

In the second model, the portal team is expected to: *a*) create a separate space on the portal for a strategically selected field or domain of research—with a focus, initially, on one geographic area/hotspot of concern; *b*) make the data accessible (while respecting confidentiality, security, intellectual property rights, etc.) to those who want to use it by putting it on the portal’s own servers and/or by enabling access to it via a participant’s external server through federated (collaborative grid-enabled) arrangements; and *c*) provide tools for secure data archiving, sharing, integration, visualization, and analysis. The emphasis in this second model is to enable a network of scientists to collectively develop a data grid in a mutually defined area of concentration (i.e., field or domain, as opposed to particular project) that would be beyond the capacity of any one group to build and maintain on their own. This data grid would then become a collective resource for multiple and diverse projects.

As an example of the second model of portal applications, the portal team is developing a customized environment to support basic and applied studies of contaminated sediments. Since early September 2005, the U.S. EPA has collected approximately 1,800 sediment and soil samples from over 430 sites in Jefferson, Orleans, Plaquemines, and St. Bernard Parishes (parishes that were flooded with > 3 m of water from Lake Pontchartrain and the Mississippi River/Gulf of Mexico outlet). Most of the samples, as noted in the U.S. EPA’s most recent findings on this matter (U.S. EPA 2006), were analyzed for > 200 metals and organic chemicals. The U.S. EPA collaborated with many agencies in this process, including the Louisiana Department of Environmental Quality, the CDC, the Agency for Toxic Substances and Disease Registry, the Louisiana Department of Health and Hospitals, and FEMA. According to the U.S. EPA’s final report (U.S. EPA 2006),

The sample results indicate that, in general, the sediments left behind by the flooding from the hurricanes are not expected to cause adverse health impacts to individuals returning to New Orleans.

However, the same report indicates that hot spots of concern do exist. Some of the sites sampled showed elevated levels of arsenic, lead, benzo[*a*]pyrene, and diesel and oil range organic petroleum chemicals. Besides the U.S. EPA, other groups including environmental non-governmental organizations, have also collected contamination data and have expressed strong environmental health concerns. To support integration of sediment sampling data from different sources with hurricane damage and potential exposure routes, the portal hosts the “sediments” research space and an online GIS. The system is being designed to enable analysis of spatial patterns in contaminated sediments and help prioritize selection of additional sampling sites, as well as to provide relevant information for risk assessment, risk communication, and remediation. Both of the examples cited above exemplify the flexibility of the NIEHS Portal.

## Results

Whether considering the public portion of the portal or applications developed under either of the two models, the NIEHS Portal provides the ability to

- Create collaborative workspaces quickly and easily (e.g., document and data set exchange, discussion groups, forums) and publish them online for use by internal and external user communities
- Facilitate structured and secure information exchange and knowledge sharing between collaborators, regardless of their physical location (users will be able to place their data sets in a secure managed environment, where, for example, these data sets can be shared with collaborators or discovered on search requests)
- Use advanced information management, analysis, and visualization applications, and connect different applications in personal research workflows
- Enable search and query to multiple distributed and separately maintained databases (to alleviate duplication and remove data maintenance hassles, the different databases can be accessed via a single log-on mechanism, thereby removing the hassle of logging into each database separately and thus providing an easier platform for integrating disparate information)
- Synchronize user information from existing directories [i.e., MyProxy, LDAP (Lightweight Directory Access Protocol)]
- Within the same system, receive access authorization to computing resources that are normally not available to regular web users, such as supercomputers, clusters, or large data archives.

The NIEHS Portal is evolving as a system that enables multidisciplinary collaboration, including public–private sector partnerships, through two forms of integration: first, integration of science to science through the joining of scientific perspectives, data, knowledge, and approaches across disciplines; and second, integration of science to society by creating pathways to move research and technology into contexts where they are most needed for decision making, problem solving, and innovation. Several goals spelled out in the 2006–2011 NIEHS Strategic Plan (NIEHS 2006c) emphasize the importance of these two forms of integration: Goal III calls for enabling integrative and cross-cutting research approaches; Goal IV calls for improving and expanding capacity for community-linked research; and Goal VII underscores the importance of supporting development of partnerships. Other types of integration are also important. The portal can be used to integrate data and information to allow views across temporal and spatial scales. It also has potential to integrate models used in societal and scientific communications—thereby enabling interaction across model-derived (rather than observational) data.

### User-needs aassessment, system evaluation, and feedback

To identify and assess the needs of the NIEHS Portal user community, as well as to test system performance with a diverse range of users, a series of meetings and public workshops were conducted in the Gulf Coast Region in April and May of 2006. The findings of the NIEHS Portal User Community Development effort were reported previously (Pezzoli et al. 2006). The NIEHS Portal user community stakeholders identified as part of this process included local, regional, and federal government agencies (Louisiana Department of Environmental Quality, planning agencies, U.S. EPA, FEMA, etc); researchers; non-governmental organizations, and the general public. The user community expressed an interest in seeing the portal populated with pre- and post-Katrina data sets on air quality, soil and sediment quality, surface and groundwater quality, health outcomes, demographics, National Pollutant Discharge Elimination System permits, flood data, rebuilding plans, building permits, and overall data sets having greater spatial and temporal representation. In addition, to the immediate and long-term environmental health research applications of the portal, the user community expressed foreseeable applications in evacuation studies, damage-assessment studies, landscape and wildlife studies, and inundation vulnerability and risk studies, among others. Comments from the user community regarding the portal centered around making the system more “user friendly”, including developing front-end interfaces based on applications such as Google Map (http://map.google.com/) and/or Google Earth (http://earth.google.com/). Also, other suggestions included adding inventories of existing research projects in the region, linking technical reports to map locations, providing interactive map generation, providing dynamic data entry tools, and providing clear and concise environmental health information designed for broad audiences in different languages as well as a mechanism for user feedback. Much of the feedback was used to generate the first version of the portal, with other functionalities and features to be added in the future. The present system is intended to serve the Gulf Coast Region, but it is anticipated that similar systems could be built for diverse applications involving larger or smaller spatial scales.

## Conclusions

The NIEHS Portal combines advances in GIS, data integration, and visualization technologies through new forms of grid-based cyberinfrastructure. A core component of the portal is an online interactive GIS that can seamlessly integrate disparate sources of comparable environmental health data from distributed databases, enabling visualization and analyses of geographic relationships, as well as modeling of contaminant transport, to help identify environmental hazards to human health.

A key advantage of the NIEHS Portal is that it enables the collaborative and multidisciplinary research needed to address complex environmental health issues by providing a common data exchange platform and workbench. The NIEHS Portal environment provides members of the research community with a flexible framework in which to collaborate with research partners and conduct both analyses and visualization using a variety of data sets and web-based tools. Parts of the portal are open to the public, while others are reserved for individual and project research spaces. The secure area of the portal can be used by researchers to upload and integrate their own data into the existing data sets available within the portal, which can then be viewed or manipulated by other members of their defined research group. Customized tools for a particular project can also be built into the portal, allowing researchers to utilize common applications among collaborators while preserving security throughout. This collaborative workspace for researchers is intended to be a tool that can help link environmental health projects without each research group having to set up and maintain a variety of data sets and information technology infrastructure.

The NIEHS Portal facilitates information and knowledge sharing because it eliminates the need for each research group to design, build, and maintain its own collaborative system. For the NIEHS, this effort is in line with one of its priority funding and emphasis areas (i.e., infrastructure) and also supports the critical challenge of enabling integrative research. In addition to serving a critical need in disaster response and recovery, this effort serves as a prototype for environmental health science research in the modern scientific and computing era. At a global scale, many of the world’s technology leaders (e.g., Europe, Asia) have begun to invest in cyberinfrastructure technology as part of a strategy to provide their scientists with the tools to deal with increasing amounts of data and information being generated by large-scale projects while accelerating the production and sharing of knowledge and its benefits to society. However, advances in technology and the availability of powerful tools alone will not suffice if both a culture and incentives for multidisciplinary collaboration are not in place and systems are not built with user needs in mind (Buetow 2005).
